# French validation of the Brace Questionnaire (BrQ)

**DOI:** 10.1186/s13013-017-0126-y

**Published:** 2017-06-12

**Authors:** Julie Deceuninck, Aurélie Tirat-Herbert, Nuria Rodriguez Martinez, Jean-Claude Bernard

**Affiliations:** 1 Laboratoire de Physiologie de l’Exercice, Saint-Etienne, France; 2 CMCR des Massues – Croix Rouge française, Lyon, France

**Keywords:** Brace Questionnaire, Quality of life, Brace, Idiopathic scoliosis

## Abstract

**Background:**

Quality of Life (QoL) scales have to be introduced in the treatment evaluation of our patients with adolescent idiopathic scoliosis.

Vasiliadis et. al. created the Brace Questionnaire (BrQ), which is specific for brace-treated adolescents. This tool was developed and validated in Greek.

The aim of our study was to undertake the process of cultural adaptation of the Brace Questionnaire (BrQ) into French.

**Methods:**

The BrQ is made of 34 items on Likert scale, divided in eight domains. The questionnaire was developed for self-completion by the children and is adapted for 9 to 18-year-old patients.

The process of cultural adaptation of the questionnaire was in accordance with the International Quality of Life Assessment (IQOLA) guidelines.

In the first place, descriptive statistics were used to calculate mean scores and standard deviations for a given question and a domain. The second level was comparative, concerning reliability and validity.

**Results:**

The internal consistency was satisfactory; Cronbach’s alpha coefficient was 0.85. There were no floor or ceiling effects.

**Conclusions:**

The French version of the BrQ (F-BrQ) is reliable and reproducible, and can therefore be used to evaluate the quality of life of children and adolescents treated with a brace for idiopathic scoliosis.

**Electronic supplementary material:**

The online version of this article (doi:10.1186/s13013-017-0126-y) contains supplementary material, which is available to authorized users.

## Background

It has long been a practice for physicians to take patients’ quality of life into account when evaluating the impact of their interventions. Practically speaking, evaluating the patient’s quality of life is routine: it is a natural component of the doctor-patient relationship. It is the famous “How are you feeling?”, but the practice is informal and intuitive, and lacks rigour.

There are various questionnaires in France used to evaluate the quality of life of scoliosis patients (SRS-22 SF-36) [1, 2], but they are not aimed specifically at children and adolescents who have to wear a brace as part of their treatment, and are intended only for patients whose treatment has come to an end.

In 2006, Vasiliadis et al. created the Brace Questionnaire (BrQ), which is aimed specifically at children and adolescents undergoing orthopaedic treatment for scoliosis. The questionnaire was developed and validated in Greek [3].

The aim of this study was to adapt the BrQ to the French language and culture.

## Methods

The Brace Questionnaire [3] comprises 34 Likert-scale items associated with eight domains:

- General health perception
- Physical functioning
- Emotional functioning
- Self-esteem and aesthetics
- Vitality
- School activity
- Bodily pain
- Social functioning

The questionnaire was developed so that children and adolescents between the ages of 9 and 18 could answer the questions on their own. Scoring of the BrQ is as follows:

- For items 4, 5, 6, 12, 14, 15, 16 and 17, “Always” receives a score of 5, “Most of the time” receives a score of 4, “Sometimes” receives a score of 3, “Almost never” receives a score of 2 and “Never” receives a score of 1.
- For items 1, 2, 3, 7, 8, 9, 10, 13, 18, 19, 20, 21, 22, 23, 24, 25, 26, 27, 28, 29, 30, 31, 32, 33 and 34, “Always” receives a score of 1, “Most of the time” receives a score of 2, “Sometimes” receives a score of 3, “Almost never” receives a score of 4 and “Never” receives a score of 5.

Each item score is then multiplied by 20, and the total score is divided by 34. The minimum score is therefore 20, and the maximum is 100. A higher score indicates better quality of life. A subscale score can be calculated for each of the eight domains by dividing the total score of each dimension by the number of items it comprises.

### Adaptation process

For the transcultural adaptation of the BrQ, we followed the IQOLA guidelines [4, 5].

The BrQ was translated from Greek to French by two translators certified by the Greek Embassy in Lyon. One of the two specialized in medical translation, the other did not. The two translators reached a consensus with a medical specialist and a physiotherapist specializing in scoliosis, and a preliminary version of the French questionnaire was established.

Two other certified translators (again one specialized in medical translation, the other not) then translated the preliminary version of the questionnaire from French to Greek. The four translators, the medical specialist and the physiotherapist reached a consensus on the consistency of the translated Greek version with the validated version of the BrQ and established a final preliminary version of the French BrQ (F-BrQ).

Forty patients were included in the study. Each one filled out the F-BrQ once then again 1 week later. The parents and children were provided with verbal and written information, and signed a consent form. All of the patients were treated by the same medical specialist and wore the same brace (Carbon Brace).

### Inclusion criteria

- Age 9 to 18 years
- Wearing a brace for more than 12 h a day for more than 3 months
- Cobb angle between 18° and 40°
- Thoracic thoracolumbar or double major curve

### Data gathered

- Sex
- Age at the time the questionnaire was filled out
- Weight
- Height
- Number of months wearing the brace
- Number of hours a day wearing the brace
- Curve pattern
- Cobb angle

### Statistical analysis

The statistical analysis was carried out using XLStat software (Addinsoft). The Shapiro-Wilk test was used to verify the normal distribution. First we used descriptive statistics to describe the population and calculate the mean and standard deviation for each item and each domain.

Then we compared the reliability and validity of the data. The two most important aspects for reliability are consistency and stability. Internal consistency was measured using Cronbach’s alpha coefficient. Kendall’s tau coefficient was used for the test-retest to measure temporal stability. To reduce the memory effect, the second test was administered after a 7-day interval. In terms of validity, the BrQ was assessed for floor and ceiling effects. The distribution of results would indicate the number (percentage) of patients with a minimum score (floor effect) and the number (percentage) of patients with a maximum score (ceiling effect).

## Results

This study involved 40 patients 36 girls (90%) and 4 boys (10%) whose characteristics are described in Table 1.

**Table 1:** Description of the study subjects

	Mean (SD)	Min.	Max.
Age (years)	12.72 (1.9)	9	17
Body weight (kg)	45.8 (8.4)	28	63
Height (cm)	157.2 (10.0)	138	184
Cobb angle (degrees)	24.4 (5.6)	18	35
Duration of wearing brace (months)	19.5 (18.1)	3	72
Average time spent in brace per day (hours)	17.5 (3.9)	10	22

Curve pattern: 54.3% double major, 34.3% thoracic, 5.7% thoracolumbar and 5.7% double thoracic.

The mean minimum and maximum scores obtained on the BrQ are presented in Table 2.

**Table 2:** Distribution of mean, minimum and maximum scores of French BrQ

Questionnaire	N	Min.	Max.	Mean	SD
First trial	40	49	92	76.0	10.5
Second trial	32	51	94	73.8	11.1

Table 3 compares the values of Cronbach’s alpha and Kendall’s tau coefficients for the French BrQ with Greek and Polish values.

**Table 3:** The value of Cronbach’s alpha and Kendall’s tau coefficients

Questionnaire	French version		Polish version		Greek version (original)	
	Cronbach alpha	Kendall tau	Cronbach alpha	Kendall tau	Cronbach alpha	Kendall tau
BrQ	0.85	0.79	0.94	0.82	0.82	–

Table 4 presents the mean standard deviation, minimum and maximum scores and floor and ceiling effects for each domain of the F-BrQ.

**Table 4:** Minimum and maximum scores, mean, standard deviation and floor and ceiling effects for each BrQ domain

BrQ domain	Number of items	Min.	Max.	Mean	SD	Floor effect (%)	Ceiling effect (%)
General health perception	2	4	10	7.4	1.8	0 (0)	6 (15)
Physical functioning	7	19	35	26.9	4.0	0 (0)	1 (2.5)
Emotional functioning	5	8	25	17.5	4.7	0 (0)	1 (2.5)
Self-esteem and aesthetics	2	2	10	6.8	1.8	1 (2.5)	2 (5)
Vitality	2	2	9	6.4	1.9	1 (2.5)	0 (0)
School activity	3	8	15	12.9	2.0	0 (0)	10 (5)
Bodily pain	6	9	30	23.2	5.0	0 (0)	2 (5)
Social functioning	7	17	30	29.1	4.3	0 (0)	1 (2.5)

We did not find a floor or ceiling effect for the total F-BrQ score either time the questionnaire was filled out.

## Discussion

### Statistical significance

Cronbach’s alpha coefficient is a statistical indicator that varies between 0 and 1 and that makes it possible to assess the homogeneity (the internal consistency of an evaluation or measurement instrument made up of an assembly of items designed to measure a single “underlying” construct (or dimension)): the level of knowledge or skills in a given area aptitude, attitude, motivation or interest in a given domain or object, etc. The closer Cronbach’s alpha coefficient is to 1, the greater the degree of homogeneity (internal consistency) of the items. Practically speaking, the internal consistency of an instrument is satisfactory when the coefficient is at least 0.80 [6]. The French version of the BrQ has a higher Cronbach’s alpha coefficient (0.85) than the recommended minimum, which means that it is both consistent and reliable (Additional file 1). The Cronbach’s alpha coefficient for the original Greek version is 0.82, for the Polish version, 0.94, and for the Italian version, 0.86, which confirms the BrQ’s reliability [7, 8].

Kendall’s tau coefficient is a statistic that measures the association between two variables. More specifically, it is a measure of rank correlation between two variables. The closer Kendall’s tau coefficient is to 1, the greater the correlation. Practically speaking, the internal consistency of an instrument is generally considered satisfactory when the coefficient is at least 0.70 [9, 10]. We used Kendall’s tau coefficient to measure temporal stability. Kendall’s tau coefficient for the French BrQ is 0.79. The F-BrQ is therefore considered to be stable over time, like the Polish version (*r* = 0.82) and the Italian version (*r* = 0.88).

Like the Greek and Polish researchers, we did not find a floor or ceiling effect for the total F-BrQ score.

Orthopaedic treatment for scoliosis is lengthy and restrictive and the announced or anticipated results must take into account the clinical, radiological and cosmetic impact of the condition.

The diagnosis of scoliosis in a child or adolescent is serious as is the introduction and monitoring of conservative treatment.

- Patients who are given a brace to wear will have to wear it for months or even years because of the continued growth of the spinal column. Few treatments other than those for tumours or trauma require such long-term commitment on the part of the patient. The duration of the orthopaedic treatment is implicit in the moral contract between the patient, his or her family and the physician, and must be taken into account in the measurement of impact on quality of life.
- The number of hours a day that the patient must wear the brace is also discussed: the physician will have to motivate the patient and his or her family and sometimes even adapt the protocol to take family and school events into account in an effort to preserve the quality of life of everyone involved.

The concept of treatment is closely related to two other concepts: duration of treatment which should be as short as possible, and cure. Consider, for example, a pain syndrome for which the physician can prescribe fast-acting and effective analgesics.

- Treating scoliosis with a brace is restrictive and does not necessarily bring about healing within the strict meaning of the term. The physical constraint of the brace varies depending on the model and the type and severity of the deformity determined based on 3D analysis. The brace supports certain areas of the torso without causing pain, but the impact of the “agonizing restriction” is discussed with the patient in order to minimize its effect on quality of life.
- Very few cases of scoliosis are ever cured, and physicians cannot tell patients that they will be able to rid themselves of the condition based on scientific fact. Generally speaking, the patient can expect scoliosis to stop getting worse or for its clinical and radiological impact to lessen with conservative treatment. Adolescents are usually more preoccupied by the cosmetic aspect of scoliosis, and ask questions about their condition as well as their future. The fact that there is no cure causes psychological strain related to the risk of relapse in adulthood that could affect the patient’s social, family and work life.

Treating scoliosis using a brace should not be limited to the clinical and radiological aspects of the deformity but should also include therapeutic education concerning quality of life throughout the treatment period and beyond. This holistic approach to the patient ensures a better understanding of the different steps in the treatment and a full compliance of the patient and those around him or her.

Quality of life in patients with scoliosis is usually studied using specific questionnaires [11]. The most common is the one developed by the Scoliosis Research Society (SRS) which evaluates specific problems related to scoliosis and provides a wellness overview. The SRS-22 questionnaire has been translated and validated in several languages [1, 12–21]. However, it does not evaluate the impact of wearing a brace on quality of life.

There is also the Bad Sobernheim Stress Questionnaire mit Korsett (BSSQ-K) published by Botens-Helmus [22]. It is not specific to children and does not raise family-, school- and activity-related issues or problems actually caused by the brace.

The BrQ is the first questionnaire that specifically evaluates the quality of life of scoliotic children and adolescents who wear a brace.

## Conclusions

The French version of the BrQ (F-BrQ) is reliable and reproducible, and can therefore be used to evaluate the quality of life of children and adolescents treated with a brace for idiopathic scoliosis.

## Additional file

Additional file 1: French version of the BrQ (F-BrQ). (DOC 132 kb)

## Abbreviations

BrQ: Brace Questionnaire
BSSQ-K: Bad Sobernheim Questionnaire mit Korsett
F-BrQ: French Brace Questionnaire
IQOLA: International Quality of Life Assessment
QoL: Quality of life
SRS: Scoliosis Research Society
