# Supplementary material for: French validation of the Brace Questionnaire (BrQ)
Source: Scoliosis Spinal Disord. 2017 Jun 12;12:18. doi: 10.1186/s13013-017-0126-y (PMC5467049; doi:10.1186/s13013-017-0126-y)
Supplement: Additional file 1: — French version of the BrQ (F-BrQ). (DOC 132 kb) [file 13013_2017_126_MOESM1_ESM.doc]

**BRACE** **QUESTIONNAIRE**

**Version française (BrQ-F)**

***Le questionnaire ci-dessous comporte des questions concernant tes réflexions et ton ressenti au sujet de ta santé. Il ne s'agit pas d'un test, il n'y a ni bonne ni mauvaise réponse.***

- ***Lis attentivement chaque question***
- ***Choisis la réponse qui te semble la mieux te correspondre et***

***mets une* x *sur le carré correspondant***

| ***Exemple*** | **Jamais** | **Rarement** | **Quelques fois** | **Souvent** | **Constamment** |
| --- | --- | --- | --- | --- | --- |
| **Au cours de la semaine dernière j'ai eu envie de lire** |  |  |  | **x** |  |

**Donne-nous, s’il te plaît, des informations sur toi-même**

**Tu es : Une Fille Un Garçon Âge : ………. ans**

**Date …………………………..**

| **Au cours des 3 derniers mois…** | **Jamais** | **Rarement** | **Quelques fois** | **Souvent** | **Constamment** |
| --- | --- | --- | --- | --- | --- |
| **Le corset te donnait-il l'impression d'être malade ?** |  |  |  |  |  |
| **As-tu peur que ta scoliose s'aggrave ?** |  |  |  |  |  |

| **Au cours des 3 derniers mois…** | **Jamais** | **Rarement** | **Quelques fois** | **Souvent** | **Constamment** |
| --- | --- | --- | --- | --- | --- |
| **Quand tu marchais, te sentais-tu fatigué à cause du corset ?** |  |  |  |  |  |
| **Pouvais-tu courir avec le corset ?** |  |  |  |  |  |
| **Mettais-tu le corset seul ?** |  |  |  |  |  |
| **Enlevais-tu le corset seul ?** |  |  |  |  |  |
| **Ne pouvais-tu pas manger correctement car tu portais le corset ?** |  |  |  |  |  |
| **Ne pouvais-tu pas bien dormir à cause du corset ?** |  |  |  |  |  |
| **Avais-tu des difficultés pour respirer ?** |  |  |  |  |  |

| **Au cours des 3 derniers mois…** | **Jamais** | **Rarement** | **Quelques fois** | **Souvent** | **Constamment** |
| --- | --- | --- | --- | --- | --- |
| **Le corset te rendait-il nerveux ?** |  |  |  |  |  |
| **Te sentais-tu triste à cause du corset ?** |  |  |  |  |  |
| **Te sentais-tu heureux ?** |  |  |  |  |  |
| **Crois-tu que ta vie serait meilleure si tu ne portais pas le corset ?** |  |  |  |  |  |
| **Crois-tu que la thérapie avec le corset a été bénéfique pour toi ?** |  |  |  |  |  |

| **Au cours du mois précédent…** | **Jamais** | **Rarement** | **Quelques fois** | **Souvent** | **Constamment** |
| --- | --- | --- | --- | --- | --- |
| **Te sentais-tu fier de toi ?** |  |  |  |  |  |
| **Étais-tu satisfait de toi-même ?** |  |  |  |  |  |

| **Au cours du mois précédent…** | **Jamais** | **Rarement** | **Quelques fois** | **Souvent** | **Constamment** |
| --- | --- | --- | --- | --- | --- |
| **Te sentais-tu fort et plein d'énergie ?** |  |  |  |  |  |
| **Te sentais-tu fatigué et épuisé ?** |  |  |  |  |  |

| **Au cours du mois précédent…** | **Jamais** | **Rarement** | **Quelques fois** | **Souvent** | **Constamment** |
| --- | --- | --- | --- | --- | --- |
| **Avais-tu des difficultés pour tes cours à cause du corset ?** |  |  |  |  |  |
| **Étais-tu absent de l'école à cause du corset ?** |  |  |  |  |  |
| **Étais-tu distrait en classe à cause du corset ?** |  |  |  |  |  |

| **Au cours du mois précédent…** | **Jamais** | **Rarement** | **Quelques fois** | **Souvent** | **Constamment** |
| --- | --- | --- | --- | --- | --- |
| **Prenais-tu des médicaments car tu avais mal ?** |  |  |  |  |  |
| **Avais-tu mal la nuit ?** |  |  |  |  |  |
| **Avais-tu mal quand tu marchais ?** |  |  |  |  |  |
| **Avais-tu mal quand tu étais assis ?** |  |  |  |  |  |
| **Avais-tu mal quand tu montais ou descendais des escaliers ?** |  |  |  |  |  |
| **Avais-tu des fourmillements dans les mains ou les pieds à cause du corset ?** |  |  |  |  |  |

| **Au cours du mois précédent…** | **Jamais** | **Rarement** | **Quelques fois** | **Souvent** | **Constamment** |
| --- | --- | --- | --- | --- | --- |
| **Le corset t'empêchait-il de te retrouver avec tes amis ?** |  |  |  |  |  |
| **Tes amis avaient-ils pitié de toi à cause de tes problèmes de dos ?** |  |  |  |  |  |
| **Te sentais-tu différent de tes amis car tu portais le corset ?** |  |  |  |  |  |
| **Avais-tu des problèmes avec ta famille à cause du corset ?** |  |  |  |  |  |
| **Crois-tu que tes relations avec ta famille ou tes amis auraient été meilleures si tu ne portais pas le corset ?** |  |  |  |  |  |
| **Restais-tu à la maison car tu avais honte du corset ?** |  |  |  |  |  |
| **Portais-tu des vêtements spéciaux à cause du corset ?** |  |  |  |  |  |
